# Unusual Legionnaires' outbreak in cool, dry Western Canada: an investigation using genomic epidemiology

**DOI:** 10.1017/S0950268816001965

**Published:** 2016-10-20

**Authors:** N. C. KNOX, K. A. WEEDMARK, J. CONLY, A. W. ENSMINGER, F. S. HOSEIN, S. J. DREWS, and the Legionella Outbreak Investigative Team

**Affiliations:** 1 National Microbiology Laboratory, Public Health Agency of Canada, Winnipeg, Manitoba, Canada; 2 Alberta Health Services, Calgary, Alberta, Canada; 3 O’Brien Institute for Public Health, Cumming School of Medicine, University of Calgary, Calgary, Alberta, Canada; 4 Department of Medicine, Cumming School of Medicine, Calgary, Alberta, Canada; 5 Department of Microbiology, Immunology and Infectious Diseases, Cumming School of Medicine, Calgary, Alberta, Canada; 6 Department of Pathology and Laboratory Medicine, Cumming School of Medicine, Calgary, Alberta, Canada; 7 Calvin, Phoebe and Joan Snyder Institute for Chronic Diseases, Cumming School of Medicine, University of Calgary, Calgary, Alberta, Canada; 8 Calgary Laboratory Services, Calgary, Alberta, Canada; 9 Department of Biochemistry, Department of Molecular Genetics, University of Toronto, Toronto, Ontario, Canada; 10 Public Health Ontario, Toronto, Ontario, Canada; 11 Department of Community Health Sciences, Cumming School of Medicine, Calgary, Alberta, Canada

**Keywords:** Genomic analysis, *Legionella pneumophila*, Legionnaires' disease, outbreak, ST222, Western Canada

## Abstract

An outbreak of Legionnaires' disease occurred in an inner city district in Calgary, Canada. This outbreak spanned a 3-week period in November–December 2012, and a total of eight cases were identified. Four of these cases were critically ill requiring intensive care admission but there was no associated mortality. All cases tested positive for *Legionella pneumophila* serogroup 1 (LP1) by urinary antigen testing. Five of the eight patients were culture positive for LP1 from respiratory specimens. These isolates were further identified as Knoxville monoclonal subtype and sequence subtype ST222. Whole-genome sequencing revealed that the isolates differed by no more than a single vertically acquired single nucleotide variant, supporting a single point-source outbreak. Hypothesis-based environmental investigation and sampling was conducted; however, a definitive source was not identified. Geomapping of case movements within the affected urban sector revealed a 1·0 km common area of potential exposure, which coincided with multiple active construction sites that used water spray to minimize transient dust. This community point-source Legionnaires' disease outbreak is unique due to its ST222 subtype and occurrence in a relatively dry and cold weather setting in Western Canada. This report suggests community outbreaks of *Legionella* should not be overlooked as a possibility during late autumn and winter months in the Northern Hemisphere.

## INTRODUCTION

Legionnaires' disease (LD) is a form of pneumonia caused by bacteria from the genus *Legionella*. The incubation period is typically 5–6 days but ranges from 2 to 14 days following exposure to aerosolized water containing the bacteria [1]. The first outbreak of LD was identified in July 1976, when an unknown acute respiratory disease occurred in attendees at the 58th Annual Convention of the American Legion in Philadelphia [2]. Sporadic cases of LD are typically related to exposures to aerosolized water from water-containing appliances such as air conditioners, hot tubs and humidifiers. Numerous large outbreaks of LD have been reported associated with cooling towers, which distribute aerosolized plumes to relatively large areas [3–5]. There is a general seasonality to the disease, with cases occurring more commonly during the humid and warm months from June to October in the Northern Hemisphere [6].

There have been multiple LD outbreaks reported in Canada, and they have all occurred during summer and autumn [7–9]. One of the most recent outbreaks occurred during July–September 2012 in Quebec City where 13 people died and 170 cases were reported. The source of this outbreak was found to be a water-cooling tower [7]. Another notable outbreak occurred during September–October 2005 in an Ontario long-term care facility that resulted in the deaths of 23 people and illness in another 112. The outbreak was traced to an air-conditioning cooling tower [8] and established the ST222 sequence type as a newly emergent clone whose geographical distribution has since been observed as stretching from Ontario to upper New England and the mid-Atlantic states [10, 11].

An epidemiological analysis of *Legionella* testing from Ontario (1978–2006) found 1401 cases, mainly elderly and male, and demonstrated seasonality with cases occurring in late summer to early autumn [9]. *L. pneumophila* replicates environmentally within freshwater protists [12] and has an optimal growth temperature of 35 °C [13]. When adjusted for seasonality, the incidence of sporadic human legionellosis correlates with increases in humidity [9, 14], consistent with the observation that *L. pneumophila* survival in aerosols is diminished under conditions of low relative humidity [15, 16]. Based on these observations, the prevailing weather conditions of Calgary – a generally semi-arid climate with warm and dry summer months and subzero conditions during the winter months – do not appear conducive to *Legionella* [6, 7, 9, 17]. Remarkably, we report an outbreak of LD that occurred in November and December 2012 within a specific inner-city district in Calgary, Canada during a period of subzero outdoor temperatures and low precipitation. The primary objectives of this outbreak investigation were to describe and analyse the following: epidemiology of the cases, additional case-finding measures, environmental sampling, prevailing meteorological conditions, case geomapping, microbiological testing and whole-genome sequencing of available isolates and to provide a reasonable hypothesis as to how this unusual outbreak occurred. Outbreaks with the potential to challenge our preconceived notions of LD exposure are important for modifying practices to reduce the risk of disease.

## MATERIALS AND METHODS

### Case definition, case-finding and investigative analyses

LD cases were defined as having: (1) a positive urine antigen for *L. pneumophila* serogroup 1 (LP1) within the clinical context of respiratory infection/illness; (2) symptom onset on or after 1 November 2012; (3) a history of residing/visiting/working within a 2 km radius of a Calgary inner-city district 2 weeks prior to symptom onset. No out-of-province LD cases met the case definition. A Centers for Disease Control and Prevention standardized legionellosis questionnaire [18] with minor modifications was administered to collect demographic information, illness information, and potential environmental sources. In addition, we extended case-finding efforts to identify cases that may have been exposed locally but manifested themselves outside of the province or elsewhere in the country. This standardized questionnaire provided the basis for further hypothesis generation and environmental testing. Confirmed cases admitted to intensive care were considered to have severe illness. Details of the cases including demographics, risk factors, absence of travel history and results of microbiological and serological testing were analysed using basic descriptive epidemiological techniques and presented in tabular and/or text format. To identify other potential cases, a communication was sent on 3 December 2012 to Calgary area physicians requesting them to be alert for patients with clinical signs and symptoms of *Legionella* infection and risk factors, with recommendation for laboratory testing of suspect cases.

For additional case-finding investigation, all lower respiratory tract specimens from patients from 1 October to 19 December 2012 in Calgary that were negative for influenza A/B and the Luminex respiratory virus panel were identified using the Data Integration for Alberta Laboratories (DIAL) tool. These specimens were tested for *L. pneumophila* using real-time PCR as described previously [19]. All reported LD cases in Calgary for the previous 14 years (1998–2011) were also reviewed: of 35 laboratory-confirmed cases, 33 were sporadic (unlinked) of which 22 had no history of travel outside Alberta (Alberta Communicable Disease Reporting System).

### Environmental sampling and testing

Sites for environmental sampling were chosen on the basis of the responses to the relevant sections of the standardized questionnaire pertaining to hypothesis generation and applying principles known about *Legionella* sources and knowledge of our local setting. We conducted an extensive review of potential water sources in the area of concern but because of sub-freezing point temperatures, office building cooling towers were not operational, which limited the number of sites. Water samples were acquired from household kitchen, bathroom and shower taps of confirmed cases; additional samples were taken from humidifiers where applicable. Further hypothesis-based sampling of potential community exposure sources occurred; these sites included grocery stores and water taps in office buildings frequented by confirmed cases. Samples taken from the grocery store included water specimens both distal and proximal to endpoint vegetable misters, given prior descriptions of an outbreak associated with such a source [20]. Piping from the water distribution system of the grocery store was also taken for microbial analysis. Air-conditioner systems in apartment and office buildings were not sampled since they were not functioning at that time of year. We also examined all operational cooling tower sites located within and upwind of the 2 km radius area of interest. Environmental samples were tested by cultures grown on buffered charcoal yeast extract (BCYE) and BCYE-PCV (polymyxin B, cycloheximide and vancomycin) agar which are recommended for use in the cultivation and primary isolation of *Legionella* spp. in water [21].

### Meteorological conditions

Data from historical weather records at the Calgary International Airport (Calgary, Alberta) was collected and reviewed (https://weatherspark.com/history/28433/2012/Calgary-Alberta-Canada). This is an official weather recording site in the city with records dating back to July 1955. Our primary focus was on daily recordings of temperature, precipitation, humidity, and wind speed with reporting of daily low and high readings plus means with percentile bands from the 10th–90th and 25th–75th percentiles, respectively, displayed in graphical format.

### Case geomapping

A map of the downtown Calgary area was reproduced from Google Maps (www.google.ca/maps). Searches of known construction websites in the affected sector of Calgary were conducted to identify procedures that may have produced aerosols. OpenStreetMap (www.openstreetmap.org) and Leaflet javascript libraries (www.leafletjs.com) were used to create a georeferenced map of Calgary's downtown area to which data points were added. These included the locations each individual visited (residence, business, work, social) as well as active construction sites and fire incidents during the described time period.

### Microbiological testing

Urine antigen testing and culture of respiratory specimens and blood were performed at Calgary Laboratory Services (CLS) using standard protocols [22]. Preliminary identification of isolates suggestive of *L. pneumophila* was performed using MonoFluo™ *L. pneumophila* indirect immunofluorescent antibody test kit (Bio-Rad Laboratories, Canada). All primary specimens and potentially positive culture isolates were sent to the Alberta Provincial Laboratory for Public Health for molecular testing of primary respiratory specimens and culture-positive isolates. Sequence-based typing of all PCR-positive primary specimens and culture isolates was also performed using the ESGLI protocol using a direct PCR method [23]. Culture-positive specimens were confirmed by indirect fluorescent antibody from individual colonies at the Provincial Laboratory and the isolates were sent to the National Microbiology Laboratory for serotyping [24] and whole-genome sequencing.

### Serological testing

Serum specimens collected from cases and household contacts and sent to Public Health Ontario Laboratories (Toronto) for *L. pneumophila* immunofluorescence antibody serological analysis looking for a single titre of ⩾1 : 256 or a fourfold seroconversion to ⩾1 : 128 in sequential serum samples [8].

### DNA isolation and genome sequencing

Genomic DNA was extracted from 48-h cultures [8] using the Epicentre Metagenomic DNA Isolation kit for water (Epicentre Technologies Corp., USA) and libraries were prepared using Nextera XT Sample Preparation Kit (Illumina Inc., USA). MiSeq (2 × 250 bp) sequencing was performed using 500-cycle MiSeq Reagent kits (v. 2) according to manufacturer protocols (Illumina Inc.).

### SNV analysis

Phylogenies based on single nucleotide variants (SNVs) were generated using the SNVPhyl pipeline [25]. Briefly, paired-end reads were mapped to the Toronto-2005 outbreak reference genome (Genbank accession no. CP012019) [26] using SMALT v. 0.7.6. High-quality SNVs were identified using two variant callers: FreeBayes v. 0.9.8 and SAMtools v. 1.1 mpileup (mapping and quality scores ⩾30; SNV fraction ⩾0·75; coverage ⩾15). Repetitive elements (MUMmer v. 3.23), phages (PHAST), and genomic islands (IslandViewer v. 2) [27–29] identified in the Toronto-2005 reference genome were excluded from the phylogenomics analysis. SNV loci present in all isolates were extracted and aligned. Maximum-likelihood phylogenetic trees were constructed using PhyML v. 3.1 [30] (GTR + G model, best NNIs/SPRs, initial BioNJ tree) using an approximate likelihood ratio test [31] and images were rendered in FigTree v. 1.4.1 [32].

### Genome analysis

Paired-end reads were assembled *de novo* using FLASH v. 1.2.9 [33] (overlap >20, <300) and SPAdes v. 3.50 [34] (default parameters for paired-end data; *k*mers: 21, 33, 55, 77, 99, 127). Each genome comprised 44–71 contigs and average genome coverage values between 42- and 152-fold (Supplementary Table S2). Auto-annotation using Prokka v. 1.10 [35] predicted 3239–3250 CDSs for each isolate (Supplementary Table S2). Comparative analyses were performed using MAUVE v. 2.4.0 [36] and GView Server [37].

### Accession numbers

The data for this study has been deposited at NCBI (http://www.ncbi.nlm.nih.gov/) under BioProject PRJNA291490 (BioSamples: SAMN03944915-SAMN03944919; SRA: SRR3063530-SRR3063534).

## RESULTS

### Descriptive epidemiology

**Fig. 1.**
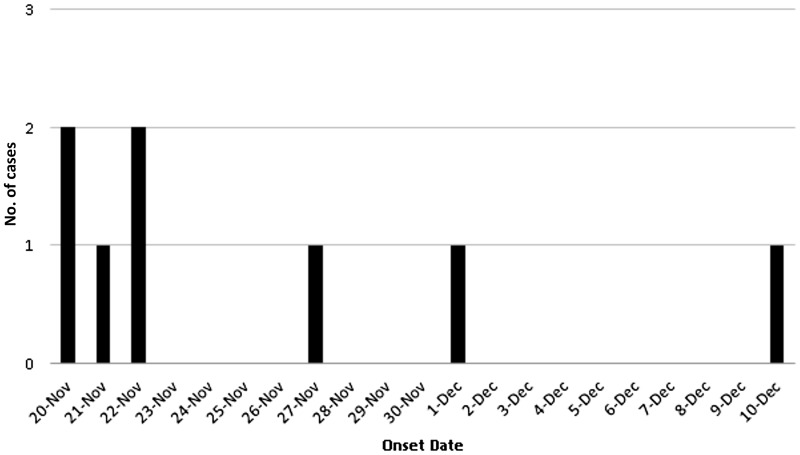
Epidemic curve. Confirmed cases of Legionnaires' disease by date of onset in Calgary, Alberta, Canada (23 November–14 December 2012) (*n* = 8).

The first five cases occurred within 48 h and with a background of an average of 2 cases/year being reported in the previous decade within Calgary, it was evident an outbreak was occurring (Fig. 1). In total, eight confirmed cases of LD were identified within a 1 km diameter area of a downtown urban sector of the city, over the time period spanning 23 November to 14 December 2012 (Fig. 1). An additional 24 potential patients admitted during this same time period were investigated but did not meet the case definition; all were *Legionella* urine antigen negative. The first case was identified on 23 November, with peak incidence occurring 26–27 November (three cases) (Fig. 1, Table 1). The average age of this case cluster was 65 years, with 50% of the patients having severe illness requiring intensive care admission. No mortalities were associated with this cluster (Table 1). Additional PCR screening of all lower respiratory tract specimens from Calgary patients from 1 October to 19 December (*n* = 189) did not identify further cases. A historical review of legionellosis cases in Calgary (1998–2011), revealed an average of 1·6 LD cases were reported annually with no history travel outside Alberta.

**Table 1.** Demographic and laboratory test results from urine antigen-positive patients from Calgary 2012 Legionnaires' disease outbreak

Case	Age (sex)	Date of diagnosis	UA test	Specimen source	Culture and IFA	LP PCR	Subtype (culture)	ST	IFA titre (acute)	IFA titre (conval.)	Critical Illness	Isolate	Accession no.
1	67(F)	23 Nov. 2012	LP1	Sputum	Neg	Pos.	n.d.	*	n.a.	n.a.	Y	n.a.	n.a.
2	51(M)	26 Nov. 2012	LP1	Sputum	Pos.: Sg1	Pos.	Knoxville	222	n.a.	2:04	N	120 824	SAMN03944917
3	71(F)	27 Nov. 2012	LP1	BAL left upper lobe	Pos.: Sg1	Pos.	Knoxville	222	n.a.	n.a.	N	120 815	SAMN03944915
4	78(F)	27 Nov. 2012	LP1	n.a.	n.a.	n.d.	n.d.	n.d.	<1:64	<1:64	N	n.a.	n.a.
5	54(M)	30 Nov. 2012	LP1	BAL right middle lobe	Pos.: Sg1	Pos.	Knoxville	222	1:256	<1:64	Y	120 825	SAMN0394491
6	65(M)	1 Dec. 2012	LP1	Aspirate	Pos.: Sg1	Pos.	Knoxville	222	n.a.	<1:64	Y	120 826	SAMN03944918
7	57(F)	8 Dec. 2012	LP1	BAL left lower lobe	Pos.: Sg1	Pos.	Knoxville	222	n.a.	1:1024	Y	120 842	SAMN03944919
8	77(F)	14 Dec. 2012	S LP1	n.a.	n.a.	n.d.	n.d.	n.d.	n.a.	<1:64	N	n.a.	n.a.

UA, urine antigen; IFA, indirect immunofluorescent assay; ST, Sequence-based typing; n.a., not available; n.d., not done; BAL, bronchoalveolar lavage;

* 6/7 alleles matched ST222 from primary specimen, unable to complete sequencing for one allelle (*NeuA*);

### Environmental sampling

In total, 42 environmental samples were collected and tested from eight residences, two office buildings and one grocery store. One positive result for *L. jordanis* was obtained from a humidifier located in the household of a case. Pipes and components of the water distribution system within the grocery store were negative for growth of *Legionella* species. Operational cooling towers located within the affected area and upwind (prevailing westerly winds) within the 2 km radius of the affected area were negative for *Legionella* growth.

### Meteorological conditions

**Fig. 2.**
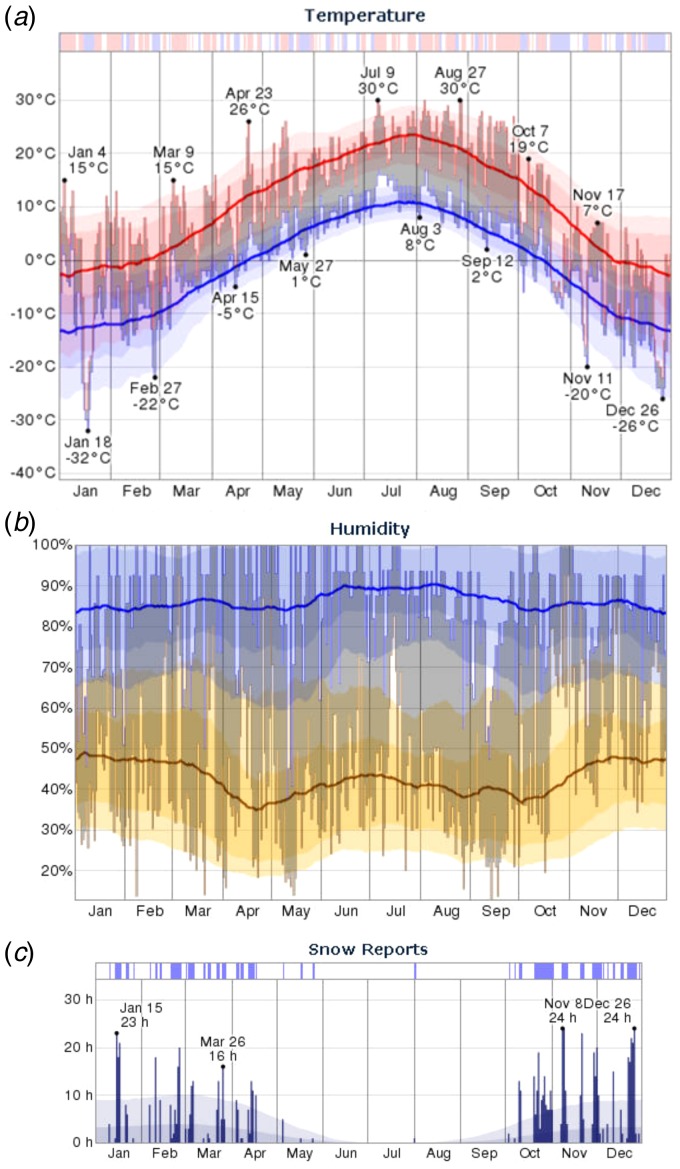
Temperature, humidity and snowfall reports for Calgary, Alberta, Canada. (*a*) The daily low (blue) and high (red) temperature during 2012 with the area between shaded grey and superimposed over the corresponding averages (thick lines) and with percentile bands (inner band, from 25th to 75th percentile; outer band from 10th to 90th percentile). The bar at the top of the graph indicates when both the daily high and low are above (red) or below (blue) average temperatures values. (*b*) The daily low (brown) and high (blue) relative humidity during 2012 with the area between shaded grey and superimposed over the corresponding averages (thick lines) and with percentile bands (inner band, from 25th to 75th percentile; outer band from 10th to 90th percentile). (*c*) The daily number of observed hourly snow reports during 2012 with normals indicated (faint shaded areas). The bar at the top of the graph is blue if there was snowfall observed that day and white otherwise.

The meterological conditions within the city of Calgary are depicted in Figure 2. The daily temperatures were typical and fluctuated between 7 °C and −20 °C in November and 6 °C and −26 °C in December (Fig. 2*a*). The most humid month of 2012 was November with an average daily low humidity of 59%, above the historical average of 45%. The average daily humidity for December was also above average (Fig. 2*b*). There was no rain recorded in this period (data not shown), but snow was reported with the largest number of recorded snow days of 2012 in December (Fig. 2*c*). The most windy month of 2012 was October with an average wind speed of 5 m/s whereas the least windy month was February, with an average wind speed of 3 m/s. Wind speeds in November and December were consistent with average recorded values (Supplementary Table S1). Typical wind direction was westerly.

### Case geomapping

**Fig. 3.**
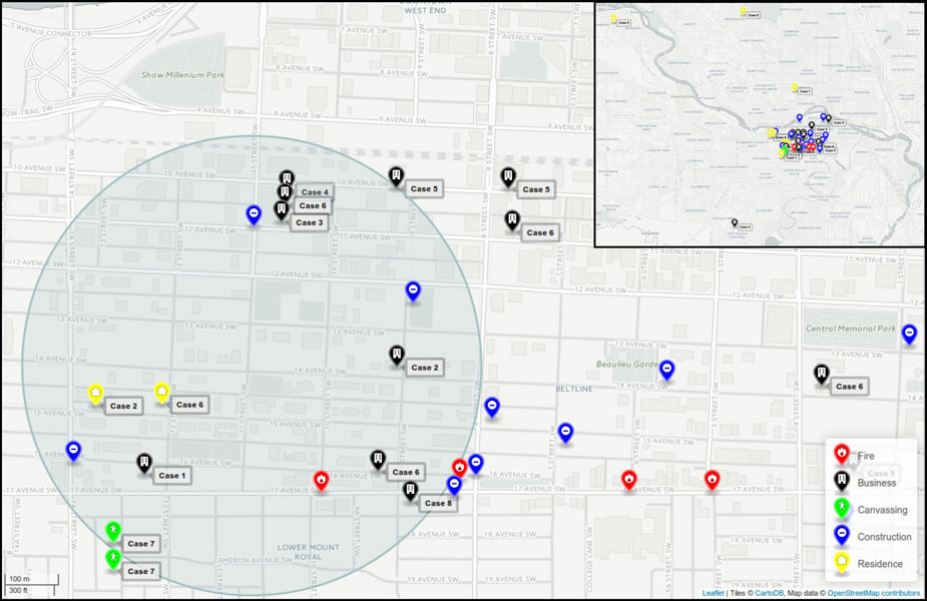
Map of downtown Calgary illustrating locations visited by Legionnaires' disease patients. The locations visited (residences, businesses, canvassing sites) by all cases in the 2012 Calgary outbreak from November to December 2012 are indicated. Active construction sites and fire incidents are also shown. A 1 km diameter zone encompasses locations visited by all eight cases in the 2012 outbreak. An interactive html map is available (https://share.corefacility.ca/index.php/s/arCfWzeT3fqNWDH).

Cases were mapped with respect to their residences within the community and their usual walking patterns based on interview data. Of the eight cases, three lived in the northwest quadrant of the city across the river and travelled or worked within the affected western ‘Beltline’ or eastern edge of the ‘Sunalta’ communities (Fig. 3); one additional case lived at the outskirts of the Beltline community. Four individuals lived within the affected community and walked within the area of interest. One individual lived across the river and travelled only by car to appointments within the affected area. All eight cases' residences or walking/travel patterns converged within a common 1 km diameter area bridging between the Beltline and Sunalta communities bordering downtown Calgary (Supplementary Fig. S1). This area included two residences (cases 2 and 6), six businesses (cases 1–6, 8), and one canvassing site (case 7) confirmed to have been visited during the potential exposure period. By visual analysis of intersections and within the 1 km diameter area, from place of residence or work or visiting, a common area of 8 × 11 blocks was identified (Supplementary Fig. S1). In total, there were 17 active construction sites and four fire incidents identified as potential water-spray sites within the area of interest during the potential exposure period.

### Microbiological testing and molecular characterization

All eight cases tested positive for LP1 urine antigen. Six were also PCR positive for *L. pneumophila* from respiratory specimens (Table 1), and 5/8 patients were culture positive from respiratory specimens. Further characterization of the five culture-positive cases identified LP1 with a Knoxville monoclonal subtype and an ST222 sequence subtype. One culture-negative patient was PCR positive for *L. pneumophila* ST222, and matched to 6/7 alleles from the other five cases confirmed to be ST222 (Table 1).

### Serological testing

Serological testing was of limited value with no cases demonstrating a fourfold rise in titre, although one case demonstrated a ⩾fourfold decrease in titres between acute and convalescent sera (Table 1) and one case had a single static titre of >1:1024. No family members of the cases were found to have either a fourfold increase or a single static high titre.

### Genomic analysis

**Fig. 4.**
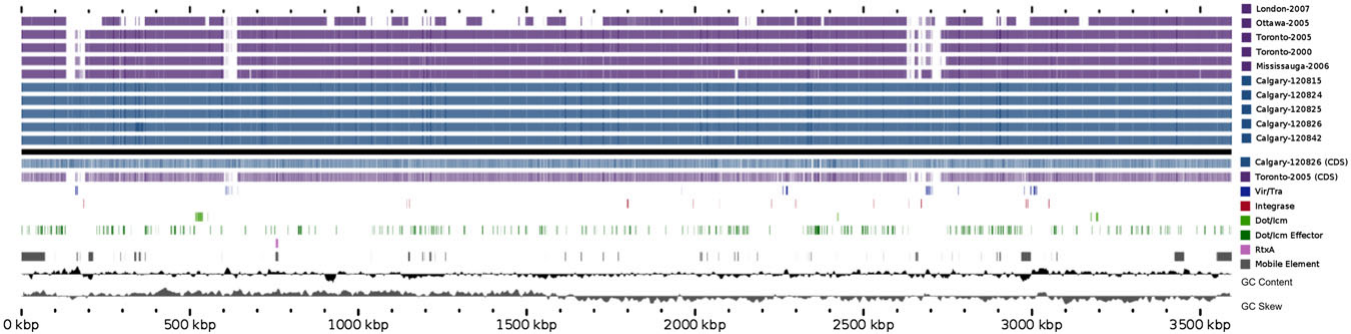
Blast Atlas of Calgary 2012 outbreak cluster isolates. A Blast Atlas was generated with GView Server using *L. pneumophila* genomes from the Calgary 2012 outbreak (turquoise) and Ontario (violet). Regions with Blast scores >80% identity and Expect values <e^−10^ to the reference genome (Calgary- 120 826) are displayed. Upper tracks: Blast analysis of draft genome sequences; lower tracks: Blast analysis of predicted CDSs and genomic elements, GC content, and GC skew. Components of the Dot/Icm system, Dot/Icm effectors, Vir/Tra homologs, Integrases, RtxA, and mobile elements (predicted by Island Viewer, PHAST, and PhiSPY) are indicated.

**Fig. 5.**
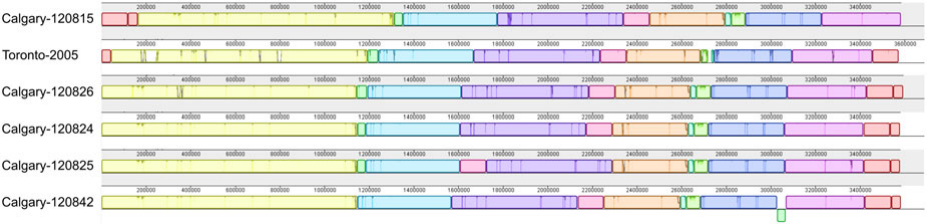
Genome alignment of *L. pneumophila* isolates showing genome architecture and synteny. The genome alignment and schematic were obtained using the Mauve software package and the CONTIGuator-generated pseudomolecules of the *de novo* assembled Calgary 2012 draft genomes. Homologous segments are illustrated as coloured blocks. Isolates 120 825 (case 5) and 120 842 (case 7) show translocated segments (pink and green, respectively) relative to the Toronto-2005 reference genome (CP012019) and to the other Calgary 2012 isolates (120 815, case 3; 120 824, case 2; 120 826, case 6).

The genomic architecture among Calgary 2012 isolates was analysed using Mauve (see Methods section). With the exception of large rearrangements observed in isolates from cases 3 and 7 (isolates 120825 and 120 842, respectively), the Calgary 2012 isolates are highly syntenic (Fig. 4, Supplementary Fig. S2). Synteny and homology in the Calgary 2012 isolates was also revealed by Blast analysis of genomic content (Fig. 5). Within the Calgary 2012 cluster, macro-level variation is limited to repetitive and multi-copy elements (such as *16S/23S* ribosomal RNA and repeats in toxin *rtxA* genes) which are known artifacts of short-read based assemblies.

**Fig. 6.**
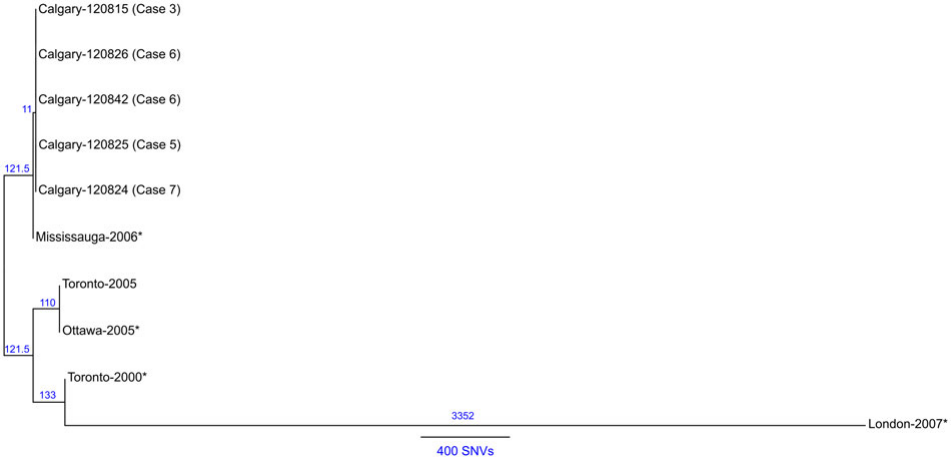
Maximum likelihood SNV phylogeny analysis of ST222 *L. pneumophila* isolates. Maximum likelihood phylogenetic model of *L. pneumophila* ST222 isolates based on 1688 core SNV loci (Supplementary Table S3) illustrating a close relationship (⩽1 SNV) among Calgary 2012 outbreak isolates (refer to Methods section). The Calgary cluster is distinguished from Ontario strains by ⩾11 core SNVs (Mississauga-2006). Strains associated with sporadic Legionnaires' disease cases are denoted with an asterisk (*). Reference genome: Toronto-2005 (CP012019). The number of SNVs between isolates according the phylogenetic model is indicated and a distance bar is shown. Calgary 2012 isolates: 120 815, case 3; 120 824, case 2; 120 825, case 5; 120 826, case 6; 120 842, case 7.

To infer the genetic distance for the Calgary 2012 isolates compared to other ST222 strains, a phylogeny based on core SNVs was generated [25] (Fig. 6). The high quality *L. pneumophila* strain Toronto-2005 reference genome [26] is representative of a clonal 2005–2006 LD outbreak linked to 135 cases including 23 deaths in Ontario, Canada [8, 10]. Analysis of the reference mapping data revealed that a single copy 17 kb subregion (Toronto-2005: 2 694 469–2 711 545) of the 77·6 kb transfer (Tra) element [26] showed allelic duplication in the Calgary-2012 isolates, coinciding with ~16 kb and ~103 kb genomic islands (Calgary- 120 826: 601 143–620 107 and 2 628 230–2 731 168). These regions show homology to several Lvh (Legionella vir homolog) and Tra components of the type IVA secretion system [38] and are flanked by hallmark elements of integrative conjugative elements (ICEs) [39]. For instance, the latter 103 kb genomic island in the Calgary- 120 826 comprises several Lvh/Tra components, integrases, and direct interspersed 45 bp tRNA^Met^ repeats (Fig. 4 and data not shown). The imperfect nature and high SNV density observed in this duplication (data not shown) indicates that at least one of these Lvh-ICE duplications was acquired through a horizontal gene transfer event. Thus, the entire region was excluded from core phylogeny analysis.

A total of 1688 high-quality SNVs were identified for the ST222 isolates (Supplementary Table S3 and Methods section). Phylogenetic SNV analysis (Fig. 6) shows that the Calgary-2012 cluster isolates form a distinct clade and are distinguished from the Ontario ST222 strains by a minimum of 11 SNVs. SNV variation within the 2012 cluster isolates is limited to ⩽1 SNV due to a single SNV in isolate 120 825 (case 5). Together, these analyses suggest that the Calgary 2012 cluster isolates are highly isogenic.

## DISCUSSION

This outbreak occurred in a relatively short time-frame within a 1 km diameter area in a densely populated inner-city area. It is notable that since *Legionella* was first described in 1976, this is the first recognized LD outbreak in Calgary. There have been confirmed LD cases within the Calgary area previously reported, including persons with no travel history outside of Alberta, although these were sporadic (unlinked). The prevailing winter weather conditions were not considered conducive to *Legionella* [6, 7, 9, 17]. Case geomapping suggested that an 8 × 11 block area was common to all the cases. Taken together, the temporality, localized geography, and isogenic isolate characterization suggests a common environmental source of exposure. The identification of the Calgary outbreak strains as ST222 is particularly noteworthy: this sequence type was first reported in Ontario in 1999 and has emerged as a frequent cause of LD in that province [10], most notably as the source of a large Toronto outbreak of disease in 2005 [8]. In addition, the occurrence of a confirmed non-travel-associated *L. pneumophila* ST222 outbreak in Western Canada is the first identified cluster of this emerging sequence type outside of Ontario and the northeastern United States [10, 11].

The structure of the duplicated Lvh-ICE region in the Calgary-2012 isolates resembles the LpcGI-2 genomic island in *L. pneumophila* strain Corby [40]. Such ICEs can be excised as part of a larger episomal element and inserted into the chromosome by a conservative cut-and-paste mechanism [40]. The imperfect nature of the Lvh-ICE duplication in Lp2012 does not support this scenario; furthermore, the lack of excessive read coverage depth at these regions in the Calgary-2012 isolates (data not shown) argue against the presence of an episomal Lvh-ICE element. Thus, while at least one of these duplicated regions was acquired via horizontal gene transfer, the mechanism (i.e. recombination due genomic island swapping or integration), requires further investigation.

Our outbreak investigation involved environmental sampling aimed at identifying hypothesized common sources, and targeted venues of common attendance for sampling. We did not identify a common source of LP1 from environmental samples, patient residences, or cooling towers within or upwind of the area of interest, and no evidence of *Legionella* infection was found in serological specimens from family members. Therefore, we hypothesize that the source of the *Legionella* strain in this outbreak may have been one or more construction or fire sites located in the area of interest. Activities such as spraying concrete or water for dust control were performed at construction sites in this area and may have created aerosol plumes. While none of the construction sites were sampled for testing, our models suggest that aerosol generating construction activities should be sampled for future suspected outbreaks of LD.

Our results are both unique and novel since they describe the occurrence of LD arising from a probable outdoor exposure and subzero temperatures which has not previously been described with *L. pneumophila*. The literature supports a seasonality of LD that occurs more frequently during summer and early autumn and in warm, humid environments [6, 7, 9, 17, 41, 42]. A large study reviewing 23 076 cases of legionellosis reported from 1990 to 2005 revealed a marked seasonality in eastern United States, with most cases reported in summer or autumn [43]. A review of legionellosis cases in central Canada also identified summer–autumn seasonality [9]. Outbreaks of community-associated or nursing-home-associated LD in Canada have generally been limited to summer and autumn months [7, 8, 44, 45] and have been associated with cooling towers. To the best of our knowledge, no published Canadian or international data have described a community-associated outbreak of *Legionella* in a cold-climate setting.

There are plausible explanations as to why this event may have occurred in a community setting with potential outdoor exposure with temperatures between 7 °C and −26 °C. As noted there were numerous active construction sites and small fire events in a localized area and temporality with frequent use of water spraying increasing the likelihood of a common exposure through aerosols. No family members showed evidence of legionellosis which argues against multiple occurrences occurring by chance alone. An interesting observation is that the relative humidity during the time of the exposures was well above the usual average which may have favoured an exposure-transmission event. Evidence has suggested a strong association with higher humidity/precipitation with a significant dose-response relationship for occurrence of cases with each of the parameters [14]. In a study of 240 legionellosis cases, despite a marked summertime seasonality, a case-crossover analysis identified a significant association with precipitation [odds ratio (OR) 2·48, 95% confidence interval (CI) 1·30–3·12] and increased humidity (OR per 1% increase in relative humidity: 1·08, 95% CI 1·05–1·11) 6–10 days before occurrence of cases [14]. In addition, although *Legionella* has been reported to grow optimally between 25 °C and 45 °C, there is evidence the bacterium is capable of sustaining growth in lower temperatures [46]. Nosocomial transmission from a cold water supply and ice machines have been reported [46], which supports cold chain transmission potential for *Legionella*.

We recognize the limitations to our study, including issues of recall bias in interviewed patients, potential for unidentified cases, the limited number of sampling sites and the absence of a confirmed environmental source. We attempted to reduce recall bias by re-interviewing patients post-recovery, as well as spouses/partners, and we looked at all lower respiratory tract specimens from patients from 1 October to 19 December 2012 in the Calgary area that were submitted for influenza testing to increase the case-finding. It is possible that with more sampling sites an environmental source may have been determined, but because of subzero temperatures cooling towers were not operational. From a genomics perspective, while we have included all available ST222 genomic data in our analyses, including additional ST222 strains may have provided additional diversity and insight. Furthermore, the *Legionella* population structure in the Calgary area is not known; thus, whether this strain was introduced or endogenous to the region of the outbreak remains an open question. Despite these limitations, the identification of nearly identical isolates of LP1 caused by a strain never reported previously in Calgary or even in western Canada, is a significant finding in our study and suggests that this was a common-source outbreak within a very limited geographical area coincident with very heavy construction activities. This outbreak has provided several important lessons and we would suggest to expand the interview process to additional family members, add questions regarding open water sources in construction areas, include more environmental sampling sites and conduct sampling at an earlier stage of the investigation.

In conclusion we present a *L. pneumophila* Knoxville ST222 outbreak with no associated mortality, but a high proportion of critical illness that mainly affected an elderly population in a setting where LD is considered very rare in terms of local acquisition. These results suggest that *L. pneumophila* may also be transmitted in a community setting in cold climatic conditions and should not be overlooked as a possibility during late autumn and winter months in the Northern Hemisphere.

## APPENDIX. *Legionella* Outbreak Investigative Team (listed alphabetically)

K. A. Bernard^1,2^, C. Berry^1^, J. Carson^3,4,5^, B. Friesen^3,6,7^, M. W. Gilmour^1,2^, M. R. Graham^1,2^, K. Houde^3^, J. MacDonald^3,6,7^, A. L. Pacheco^1^, N. Punja^3^, A. R. Reimer^1^, K. Simmonds^7,8^, R. Tellier^3,9,10^, G. Van Domselaar^1,2^, R. Zaheer^1^, A. Zetner^1^

^1^*National Microbiology Laboratory, Public Health Agency of Canada, Winnipeg, Manitoba, Canada;* ^2^*Department of Medical Microbiology, University of Manitoba, Winnipeg, Manitoba, Canada;* ^3^*Alberta Health Services, Calgary, Alberta, Canada;* ^4^*Department of Pathology and Laboratory Medicine, Cumming School of Medicine, Calgary, Alberta, Canada;* ^5^*Calgary Laboratory Services, Calgary, Alberta, Canada;* ^6^*O’Brien Institute for Public Health, Cumming School of Medicine, University of Calgary, Calgary, Alberta, Canada*; ^7^*Department of Community Health Sciences, Cumming School of Medicine, Calgary, Alberta, Canada*; ^8^*Alberta Health, Edmonton, Alberta, Canada*; ^9^*Calvin, Phoebe and Joan Snyder Institute for Chronic Diseases, University of Calgary, Calgary, Alberta, Canada*; ^10^*Department of Microbiology, Immunology and Infectious Diseases, Cumming School of Medicine, Calgary, Alberta, Canada*

## Supplementary material

For supplementary material accompanying this paper visit http://dx.doi.org/10.1017/S0950268816001965. click here to view supplementary material

## References

[1] Cunha BA, Burillo A, Bouza E. Legionnaires' disease. Lancet 2015; 387: 376–385. 26231463 10.1016/S0140-6736(15)60078-2

[2] Sanford JP. Legionnaires' disease: one person's perspective. Annals of Internal Medicine 1979; 90: 699–703. 373555 10.7326/0003-4819-90-4-699

[3] Dondero TJ Jr., An outbreak of Legionnaires' disease associated with a contaminated air-conditioning cooling tower. New England Journal of Medicine 1980; 302: 365–370. 7351928 10.1056/NEJM198002143020703

[4] Nguyen TM, A community-wide outbreak of legionnaires disease linked to industrial cooling towers –how far can contaminated aerosols spread? Journal of Infectious Diseases 2006; 193: 102–111. 16323138 10.1086/498575

[5] Ulleryd P, Legionnaires' disease from a cooling tower in a community outbreak in Lidkoping, Sweden- epidemiological, environmental and microbiological investigation supported by meteorological modelling. BMC Infectious Diseases 2012; 12: 313. 23171054 10.1186/1471-2334-12-313 PMC3536585

[6] Ng V, Laboratory-based evaluation of legionellosis epidemiology in Ontario, Canada, 1978 to 2006. BMC Infectious Diseases 2009; 9: 68. 19460152 10.1186/1471-2334-9-68 PMC2695468

[7] Lévesque S, Genomic characterization of a large outbreak of *Legionella pneumophila* serogroup 1 strains in Quebec City, 2012. PLoS ONE 2014; 9: e103852. 25105285 10.1371/journal.pone.0103852 PMC4126679

[8] Gilmour MW, Molecular typing of a *Legionella pneumophila* outbreak in Ontario, Canada. Journal of Medical Microbiology 2007; 56: 336–341. 17314363 10.1099/jmm.0.46738-0 PMC2884934

[9] Ng V, Going with the flow: legionellosis risk in Toronto, Canada is strongly associated with local watershed hydrology. EcoHealth 2008; 5: 482–490. 19370300 10.1007/s10393-009-0218-0

[10] Tijet N, New endemic *Legionella pneumophila* serogroup I clones, Ontario, Canada. Emerging Infectious Diseases 2010; 16: 447–454. 20202420 10.3201/eid1603.081689 PMC3322000

[11] Kozak-Muiznieks NA, Prevalence of sequence types among clinical and environmental isolates of *Legionella pneumophila* serogroup 1 in the United States from 1982 to 2012. Journal of Clinical Microbiology 2014; 52: 201–211. 24197883 10.1128/JCM.01973-13 PMC3911437

[12] Fields BS. The molecular ecology of legionellae. Trends in Microbiology 1996; 4: 286–290. 8829338 10.1016/0966-842x(96)10041-x

[13] Wadowsky RM, Effect of temperature, pH, and oxygen level on the multiplication of naturally occurring *Legionella pneumophila* in potable water. Applied and Environmental Microbiology 1985; 49: 1197–1205. 4004233 10.1128/aem.49.5.1197-1205.1985 PMC238529

[14] Fisman DN, It's not the heat, it's the humidity: wet weather increases legionellosis risk in the greater Philadelphia metropolitan area. Journal of Infectious Diseases 2005; 192: 2066–2073. 16288369 10.1086/498248

[15] Berendt RF. Survival of *Legionella pneumophila* in aerosols: effect of relative humidity. The Journal of Infectious Diseases 1980; 141: 689. 7373091 10.1093/infdis/141.5.689

[16] Hambleton P, Survival of virulent *Legionella pneumophila* in aerosols. Journal of Hygiene 1983; 90: 451–460. 6863914 10.1017/s0022172400029090 PMC2134264

[17] Hicks LA, Legionellosis – United States, 2000–2009. American Journal of Transplantation 2012; 12: 250–253. 22244124 10.1111/j.1600-6143.2011.03938.x

[18] United States Centers for Disease Control and Prevention. Legionellosis Hypothesis-Generating Questionnaire (http://www.cdc.gov/legionella/downloads/hypothesis-generating-questionnaire.pdf). Accessed 3 June 2016 (http://www.cdc.gov/legionella/health-depts/inv-tools-single/index.html).

[19] Fathima S, Use of an innovative web-based laboratory surveillance platform to analyze mixed infections between human metapneumovirus (hMPV) and other respiratory viruses circulating in Alberta (AB), Canada (2009–2012). Viruses 2012; 4: 2754–2765. 23202503 10.3390/v4112754 PMC3509671

[20] Mahoney FJ, Communitywide outbreak of Legionnaires' disease associated with a grocery store mist machine. Journal of Infectious Diseases 1992; 165: 736–739. 1552203 10.1093/infdis/165.4.xxxx

[21] Rice EW, (eds). Standard Methods for the Examination of Water and Wastewater, 21st edn: American Public Health Association, American Water Works Association, Water Environment Federation, 2005, pp. 9–128 to 9–129.

[22] Edelstein PH. Legionella In: Versalovic J, *et al.*, eds. Manual of Clinical Microbiology, 10th edn. Washington, DC: ASM Press, 2011, pp. 774.

[23] Mentasti M, Extension of the *Legionella pneumophila* sequence-based typing scheme to include strains carrying a variant of the N-acylneuraminate cytidylyltransferase gene. Clinical Microbiology and Infection 2014; 20: O435–441. 24245827 10.1111/1469-0691.12459

[24] Helbig JH, Pan-European study on culture-proven Legionnaires' disease: distribution of *Legionella pneumophila* serogroups and monoclonal subgroups. European Journal of Clinical Microbiology & Infectious Diseases 2002; 21: 710–716. 12415469 10.1007/s10096-002-0820-3

[25] Petkau A. SNVPhyl: Whole Genome SNV Phylogenomics Pipeline, 2015; e554b40 (https://github.com/apetkau/snvphyl-galaxy Accessed 3 June 2016.

[26] Rao C, Active and adaptive Legionella CRISPR-Cas reveals a recurrent challenge to the pathogen. Cellular Microbiology. Published online: 31 March 2016. doi:10.1111/cmi.12586. PMC5071653 26936325

[27] Zhou Y, PHAST: a fast phage search tool. Nucleic Acids Research 2011; 39: W347–52. 21672955 10.1093/nar/gkr485 PMC3125810

[28] Kurtz S, Versatile and open software for comparing large genomes. Genome Biology 2004; 5: R12. 14759262 10.1186/gb-2004-5-2-r12 PMC395750

[29] Dhillon BK, IslandViewer update: Improved genomic island discovery and visualization. Nucleic Acids Research 2013; 41: W129–32. 23677610 10.1093/nar/gkt394 PMC3692065

[30] Guindon S, New algorithms and methods to estimate maximum-likelihood phylogenies: assessing the performance of PhyML 3·0. Systematic Biology 2010; 59: 307–321. 20525638 10.1093/sysbio/syq010

[31] Anisimova M, Gascuel O. Approximate likelihood-ratio test for branches: A fast, accurate, and powerful alternative. Systematic Biology 2006; 55: 539–552. 16785212 10.1080/10635150600755453

[32] FigTree (http://tree.bio.ed.ac.uk/software/figtree). Accessed 13 November 2015.

[33] Magoc T, Salzberg SL. FLASH: fast length adjustment of short reads to improve genome assemblies. Bioinformatics 2011; 27: 2957–2963. 21903629 10.1093/bioinformatics/btr507 PMC3198573

[34] Bankevich A, SPAdes: a new genome assembly algorithm and its applications to single-cell sequencing. Journal of Computational Biology 2012; 19: 455–477. 22506599 10.1089/cmb.2012.0021 PMC3342519

[35] Seemann T. Prokka: rapid prokaryotic genome annotation. Bioinformatics 2014; 30: 2068–2069. 24642063 10.1093/bioinformatics/btu153

[36] Darling AE, Mau B, Perna NT. progressiveMauve: multiple genome alignment with gene gain, loss and rearrangement. PLoS ONE 2010; 5: e11147. 20593022 10.1371/journal.pone.0011147 PMC2892488

[37] Petkau A, Interactive microbial genome visualization with GView. Bioinformatics 2010; 26: 3125–3126. 20956244 10.1093/bioinformatics/btq588 PMC2995121

[38] Segal G, Russo JJ, Shuman HA. Relationships between a new type IV secretion system and the icm/dot virulence system of Legionella pneumophila. Molecular Microbiology 1999; 34: 799–809. 10564519 10.1046/j.1365-2958.1999.01642.x

[39] Flynn KJ, Swanson MS. Integrative conjugative element ICE-betaox confers oxidative stress resistance to *Legionella pneumophila in vitro* and in macrophages. *mBi*o 2014; 5: e01091–14. 24781744 10.1128/mBio.01091-14 PMC4010831

[40] Lautner M, Regulation, integrase-dependent excision, and horizontal transfer of genomic islands in *Legionella pneumophila*. Journal of Bacteriology 2013; 195: 1583–1597. 23354744 10.1128/JB.01739-12 PMC3624539

[41] Farnham A, Legionnaires' disease incidence and risk factors, New York, New York, USA, 2002–2011. Emerging infectious Diseases 2014; 20: 1795–1802. 25513657 10.3201/eid2011.131872 PMC4214295

[42] Biedrzycki PA, Notes from the field: increase in reported legionellosis – Milwaukee, Wisconsin, June-September 2013. Morbidity and Mortality Weekly Report 2014; 63: 63. 24452135 PMC5779433

[43] Neil K, Berkelman R. Increasing incidence of legionellosis in the United States, 1990–2005: changing epidemiologic trends. Clinical Infectious Diseases 2008; 47: 591–599. 18665818 10.1086/590557

[44] Abbas Z, Investigation of an outbreak of Legionnaires' disease in a hospital under construction: Ontario, September-October 2002. Canada Communicable Disease Report 2003; 29: 145–152. 14526691

[45] Loeb M, Two nursing home outbreaks of respiratory infection with *Legionella sainthelensi*. Journal of the American Geriatrics Society 1999; 47: 547–552. 10323647 10.1111/j.1532-5415.1999.tb02568.x PMC7166437

[46] Arvand M, Jungkind K, Hack A. Contamination of the cold water distribution system of health care facilities by *Legionella pneumophila*: do we know the true dimension? Eurosurveillance 2011; 16: 19844. 21527132

