# Supplementary material for: Unusual Legionnaires' outbreak in cool, dry Western Canada: an investigation using genomic epidemiology
Source: Epidemiol Infect. 2016 Oct 20;145(2):254–65. doi: 10.1017/S0950268816001965 (PMC5197926; doi:10.1017/S0950268816001965)
Supplement: Supplementary file 1 [file S0950268816001965sup001.zip › Supplementary_Table_S2_(Knox).docx]

**Supplementary Table S2. *De novo* Calgary 2012 *L. pneumophila* assembly metrics (Prokka)**

| **Isolate** | **# Contigs** | **Coverage (Avg depth)** | **# Bases** | **N50 length** | **%GC** | **# CDSs** | **Accession** |
| --- | --- | --- | --- | --- | --- | --- | --- |
| 120815 | 47 | 152 | 3591988 | 173758 | 38.23% | 3239 | SAMN03944915 |
| 120824 | 48 | 138 | 3589247 | 173467 | 38.25% | 3239 | SAMN03944917 |
| 120825 | 52 | 44 | 3591990 | 173758 | 38.25% | 3242 | SAMN03944916 |
| 120826 | 71 | 87 | 3613393 | 135500 | 38.24% | 3248 | SAMN03944918 |
| 120842 | 44 | 42 | 3589335 | 173653 | 38.23% | 3250 | SAMN03944919 |
