# Supplementary material for: Unusual Legionnaires' outbreak in cool, dry Western Canada: an investigation using genomic epidemiology
Source: Epidemiol Infect. 2016 Oct 20;145(2):254–65. doi: 10.1017/S0950268816001965 (PMC5197926; doi:10.1017/S0950268816001965)
Supplement: Supplementary file 1 [file S0950268816001965sup001.zip › Supplementary_Figure_legends.docx]

**Supplementary Fig. S1.** Analysis of of downtown Calgary intersections. An approximate 8 by 11 block zone of downtown Calgary was common to all Legionnaires’ disease patients based on interview data. Interactive html: https://share.corefacility.ca/index.php/s/arCfWzeT3fqNWDH.

**Supplementary Fig. S2.** CONTIGuator alignments of *de nov*o assembled Calgary-2012 draft genomes. Draft genomes for Calgary 2012 isolates (bottom tracks) were aligned to the Toronto-2005 reference genome (CP012019, top tracks) to generate pseudocontigs for MAUVE analysis and to gain structural/synteneic insights into the draft genomes (insertions, deletions, translocations). Regions of homology are depicted by red lines between the reference and the pseudoalignment. Calgary 2012 isolates: 120815, Case 3; 120824, Case 2; 120825, Case 5; 120826, Case 6; 120842, Case 7.
