# Supplementary figures and images for: Unusual Legionnaires' outbreak in cool, dry Western Canada: an investigation using genomic epidemiology
Source: Epidemiol Infect. 2016 Oct 20;145(2):254–65. doi: 10.1017/S0950268816001965 (PMC5197926; doi:10.1017/S0950268816001965)

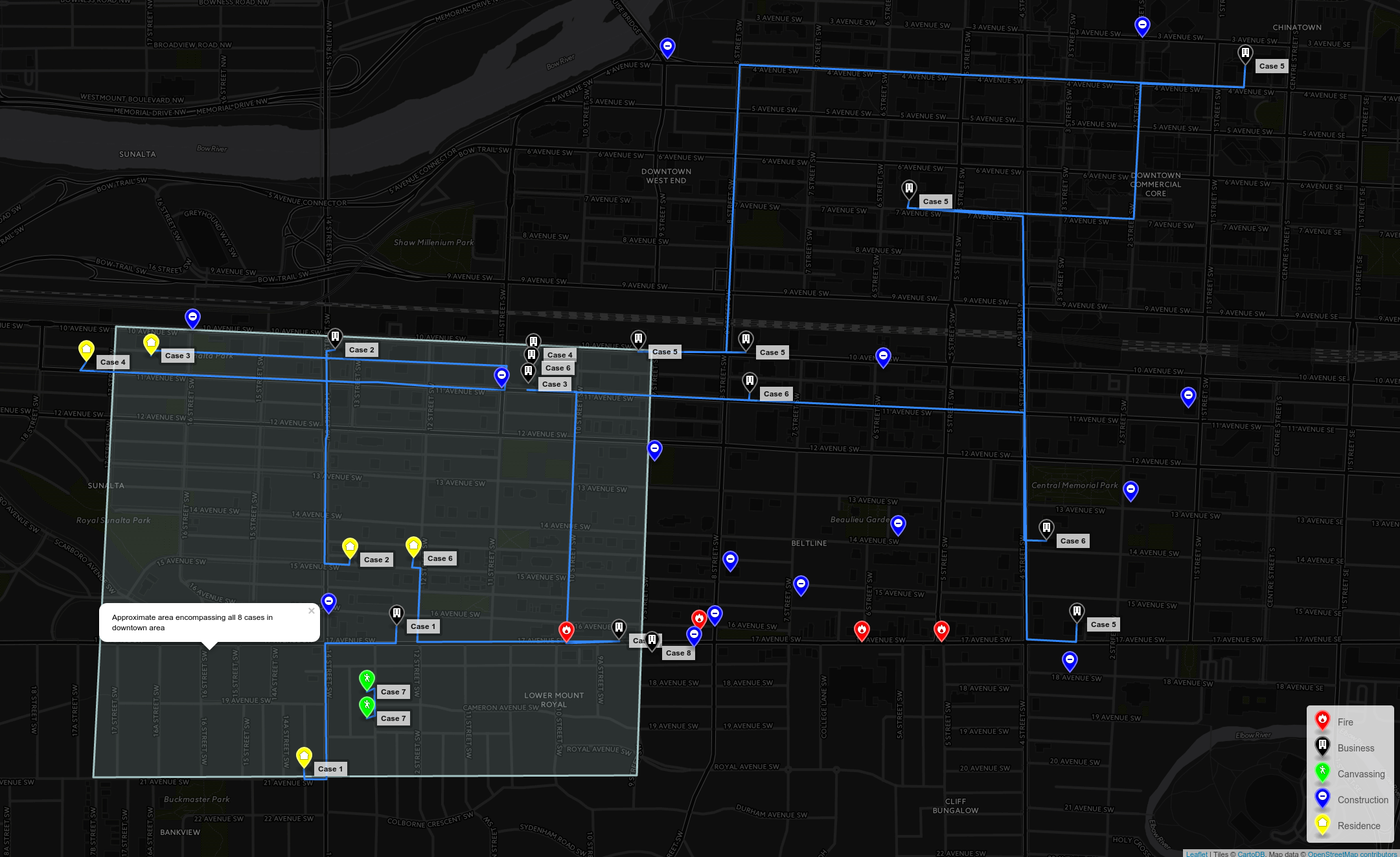

Supplement: Supplementary file 1 [file S0950268816001965sup001.zip › Supplementary_Fig._S1_(Knox).png]

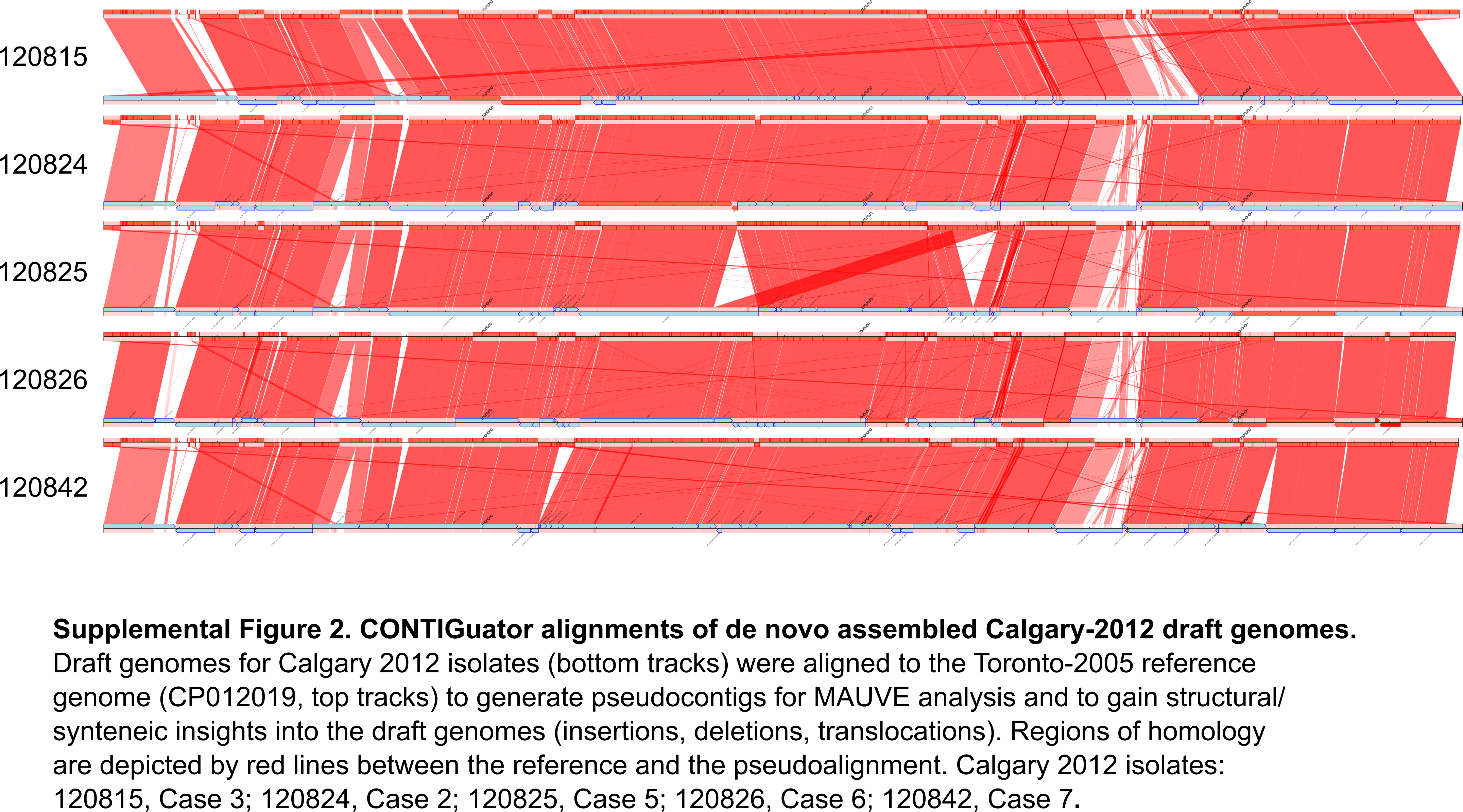

Supplement: Supplementary file 1 [file S0950268816001965sup001.zip › Supplementary_Fig._S2_(Knox).tiff]
